# Precision Agriculture and Water Conservation Strategies for Sustainable Crop Production in Arid Regions

**DOI:** 10.3390/plants13223184

**Published:** 2024-11-13

**Authors:** Yingying Xing, Xiukang Wang

**Affiliations:** Key Laboratory of Applied Ecology of Loess Plateau, College of Life Science, Yan’an University, Yan’an 716000, China; xingyingying@yau.edu.cn

**Keywords:** drought resistance, precision farming, CRISPR/Cas9, water conservation, smart agricultural IoT

## Abstract

The intensifying challenges posed by global climate change and water scarcity necessitate enhancements in agricultural productivity and sustainability within arid regions. This review synthesizes recent advancements in genetic engineering, molecular breeding, precision agriculture, and innovative water management techniques aimed at improving crop drought resistance, soil health, and overall agricultural efficiency. By examining cutting-edge methodologies, such as CRISPR/Cas9 gene editing, marker-assisted selection (MAS), and omics technologies, we highlight efforts to manipulate drought-responsive genes and consolidate favorable agronomic traits through interdisciplinary innovations. Furthermore, we explore the potential of precision farming technologies, including the Internet of Things (IoT), remote sensing, and smart irrigation systems, to optimize water utilization and facilitate real-time environmental monitoring. The integration of genetic, biotechnological, and agronomic approaches demonstrates a significant potential to enhance crop resilience against abiotic and biotic stressors while improving resource efficiency. Additionally, advanced irrigation systems, along with soil conservation techniques, show promise for maximizing water efficiency and sustaining soil fertility under saline–alkali conditions. This review concludes with recommendations for a further multidisciplinary exploration of genomics, sustainable water management practices, and precision agriculture to ensure long-term food security and sustainable agricultural development in water-limited environments. By providing a comprehensive framework for addressing agricultural challenges in arid regions, we emphasize the urgent need for continued innovation in response to escalating global environmental pressures.

## 1. Introduction

The ongoing escalation of global warming, coupled with the increasing frequency of droughts, poses a significant threat to food security. Water scarcity exacerbates the vulnerability of agricultural production in arid regions. Drylands, which comprise more than 40% of the Earth’s land area, are home to approximately 2.5 billion people and account for about 50% of livestock and 45% of global food production [1]. Faced with escalating agricultural drought conditions, the task of boosting agricultural production and ensuring regional and national food security amid resource constraints and environmental limitations has emerged as a critical concern.

Agriculture in arid regions is vital for global food security, making significant contributions to national food supplies [2]. However, these areas are characterized by ecological fragility, which includes severe soil erosion, inadequate agricultural infrastructure, and limited resilience. These challenges hinder agricultural development and restrict income growth in drylands [3]. In light of the increasing impacts of agricultural drought, particularly within the context of climate change, there is an urgent need to prioritize the enhancement of water-efficient agricultural practices and overall productivity [4]. Strengthening the resilience of agriculture in arid regions against natural disasters, while simultaneously pursuing transformations that improve quality and efficiency, is essential.

Modern agricultural technologies present promising solutions for enhancing crop drought resistance and improving water use efficiency. Strategies such as drought-resistant crop breeding, water-saving irrigation techniques, and biotechnological innovations are essential for addressing the challenges faced by agriculture in arid regions. In crop breeding, both conventional methods and molecular marker-assisted selection play significant roles in the development of drought-resistant varieties. These approaches not only enhance our understanding of the mechanisms underlying drought resistance but also facilitate the identification of the relevant genes associated with this trait [5]. Effective water management strategies, encompassing soil testing, targeted fertilization, and conservation tillage, significantly improve soil water retention and storage capacity [6]. Furthermore, advanced irrigation technologies, such as sprinkler systems, drip irrigation, and micro-irrigation, minimize water loss through evaporation, thereby ensuring a more efficient utilization of water resources [7].

**Figure 1:**
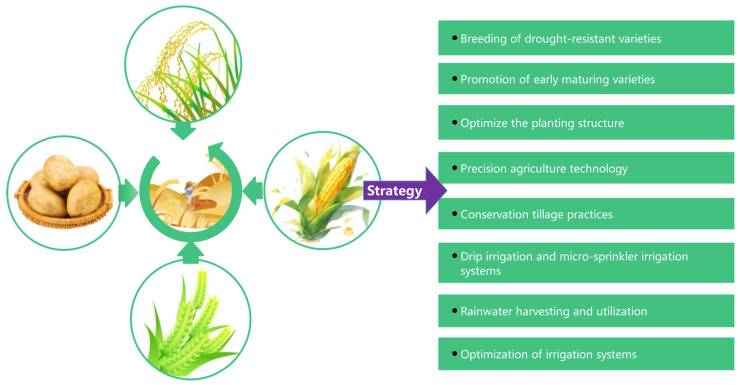
Crop breeding, efficient crop production, and agricultural water-saving technology were adopted to achieve the goal of improving the quality and efficiency of agriculture in arid regions.

Enhancing agricultural productivity in arid regions is essential for securing the global food supply and promoting sustainable farming practices. The challenges posed by limited rainfall, high evaporation rates, and severe water shortages significantly hinder efforts to increase crop yields. However, a variety of agronomic strategies, including the development of improved crop varieties, the implementation of water-efficient irrigation systems, and the adoption of effective cultivation practices, show considerable promise for boosting agricultural output in these areas (Figure 1). Modern biotechnology, which enables the cultivation of crops with traits such as drought and salinity tolerance, can substantially enhance crop adaptability and yields [8]. Furthermore, the application of water-saving irrigation technologies, such as drip irrigation under mulch, combined with integrated fertilization and water management, allows for the precise allocation of limited water resources during critical growth stages, thereby alleviating the impacts of water scarcity on crops [9].

Ensuring sustainable agricultural development in arid regions necessitates a careful balance among economic, ecological, and social benefits. A primary strategy involves enhancing the efficiency of agricultural inputs and establishing specialized farming sectors. Additionally, efforts should focus on integrating the primary, secondary, and tertiary sectors while advancing agricultural industrialization to improve overall agricultural efficiency [10]. Moreover, the adoption of conservation tillage practices, such as returning crop residues to the field, applying organic fertilizers, and planting cover crops, is crucial for enhancing soil organic matter content and improving the ecological health of agricultural land [11]. Developing water-saving agriculture, optimizing crop structures, and promoting the integration of farming and animal husbandry are crucial for achieving coordinated development [12]. By implementing these strategies, arid regions can enhance agricultural efficiency, improve ecological health, and foster coordinated development.

Enhancing agricultural productivity and sustainability in arid regions presents a significant challenge, which is further exacerbated by climate change and water scarcity. Addressing these issues necessitates a multidisciplinary approach that integrates genetic engineering, molecular breeding, precision agriculture, and innovative water management techniques. By combining advanced technologies, such as CRISPR/Cas9 gene-editing and omics technologies, with sustainable irrigation practices and soil conservation methods, we can enhance crop resilience, optimize water utilization, and improve overall agricultural efficiency. This comprehensive strategy aims to promote long-term food security and sustainable agricultural development in water-limited environments, thereby contributing to global food security and environmental sustainability.

## 2. Breeding Strategies for Drought Resistance

### 2.1. Application of Genetic Engineering in Drought Resistance Breeding

Genetic engineering plays a pivotal role in enhancing crop drought resistance by addressing the limitations of traditional breeding methods. Through molecular-level modifications, the traits associated with drought resistance can be significantly improved [13]. Transcription factors, which are essential proteins that regulate gene expression, are critical components in a plant’s response to drought [14]. By employing genetic engineering techniques, researchers can either overexpress endogenous drought-resistant transcription factor genes or introduce exogenous genes from other species to bolster a plant’s drought resistance [15]. The advent of CRISPR/Cas9 gene editing has further revolutionized drought resistance breeding by enabling precise genome editing to manipulate specific drought-related genes [16].

Drought-responsive genes, which have evolved in plants over time, are activated in response to drought stress and play a crucial role in the plant’s drought response. Numerous drought-resistant genes, including those that encode osmotic regulators, protective enzymes, and transcription factors, have been identified [17]. Through genetic engineering, these genes can be integrated into crop genomes, or negative regulatory elements can be knocked out to enhance drought resistance [18]. Furthermore, omics technologies, such as gene chips and RNA sequencing, enable researchers to screen for and identify new drought-resistant genes at the whole-genome level, thereby providing valuable genetic resources for crop breeding [19].

**Figure 2:**
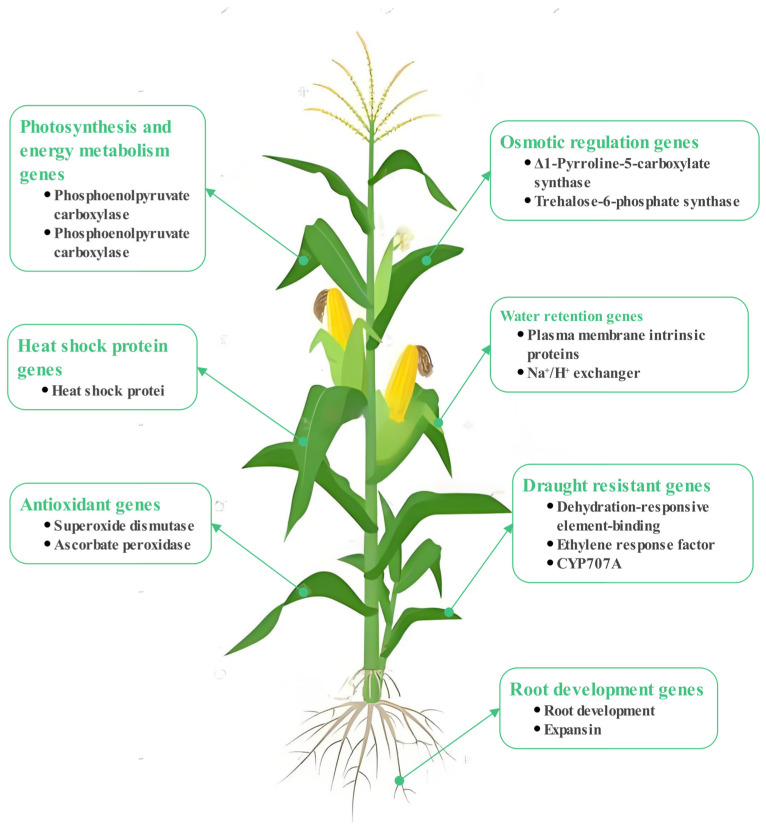
The main genes of agricultural crop breeding in arid regions were studied to enhance crop resistance to drought and other adverse conditions.

Drought-related proteins play a critical role in plant responses to drought and can serve as valuable molecular markers in breeding programs. Proteomics facilitates the identification of proteins that are differentially expressed under drought stress, thereby enabling the screening for drought-related marker proteins [20]. By incorporating genes that encode these proteins into crops, it is possible to enhance their drought tolerance (Figure 2). Furthermore, antibody technologies can be employed to develop antibodies that specifically recognize drought proteins, thereby assisting in the detection and selection of drought-resistant traits during the breeding process [21]. However, it is important to note that drought resistance is a polygenic trait that may exhibit a negative correlation with yield. Consequently, transgenic breeding for drought resistance remains largely experimental.

Future advancements in genomics, proteomics, metabolomics, and emerging technologies, such as gene editing and synthetic biology, are anticipated to significantly enhance our understanding of the mechanisms underlying drought resistance and facilitate the identification of novel drought-resistant genes. These innovations will accelerate the development of crop varieties that exhibit both drought resistance and high yields. Ultimately, the integration of conventional breeding with transgenic approaches is essential for fully leveraging the strengths of both methodologies.

### 2.2. Marker-Assisted Selection

Marker-assisted selection (MAS) is a modern breeding technique that employs the molecular markers that are linked to genes of interest to select breeding materials based on marker detection. SSR markers are particularly valued for their co-dominance, high polymorphism, and reproducibility, making them extensively utilized in the selection of drought-resistant crop traits [22]. With advancements in genome sequencing, SNP markers have emerged as essential tools in plant genetic breeding due to their abundance, wide distribution, and direct correlation with genome sequences [23]. High-density SNP chips, capable of detecting tens of thousands of SNP loci simultaneously, significantly enhance the efficiency of MAS breeding [24].

The preliminary phase in the application of molecular markers for selecting drought resistance entails the construction of a genetic map corresponding to the target traits, followed by quantitative trait loci (QTL) mapping [25]. QTLs are specific loci that influence quantitative traits. Through phenotypic identification and molecular marker analysis in segregating or natural populations, along with the application of statistical methods, researchers can identify marker intervals that are significantly associated with the target traits and estimate their contributions [26]. Once tightly linked molecular markers for drought resistance are identified, they can be employed to select and evaluate breeding materials with diverse genetic backgrounds, thereby expediting the development of drought-resistant varieties.

The basic process of MAS breeding encompasses the construction of mapping populations and the identification of drought resistance traits, the development of molecular markers, and the performance of a polymorphism analysis [27]. It also includes the construction of genetic linkage maps, the conduct of QTL mapping, and the development of markers that are closely linked to the target genes. These markers are subsequently employed to select and evaluate breeding populations [28]. Given that drought resistance is a complex quantitative trait governed by multiple genes and influenced by environmental factors, MAS breeding often integrates multiple markers and combines conventional breeding methods with field evaluations of drought resistance to enhance selection efficiency [29]. In transgenic breeding, MAS can effectively address issues such as position effects and dominance complementation, thereby improving the efficiency of introducing drought-resistant transgenes [30].

In summary, MAS provides a rapid, accurate, and reliable method for identifying drought-resistant crop genotypes. As crop genomics, bioinformatics, and sequencing costs continue to decline, the application of MAS to breeding for drought resistance is expected to become more widespread. Future research will focus on further exploring and utilizing the gene resources related to drought resistance, integrating phenotypic, genotypic, and environmental data, and establishing precise molecular design breeding systems based on multi-omics.

### 2.3. Biochemical and Physiological Mechanisms of Drought-Tolerant Crops

**Figure 3:**
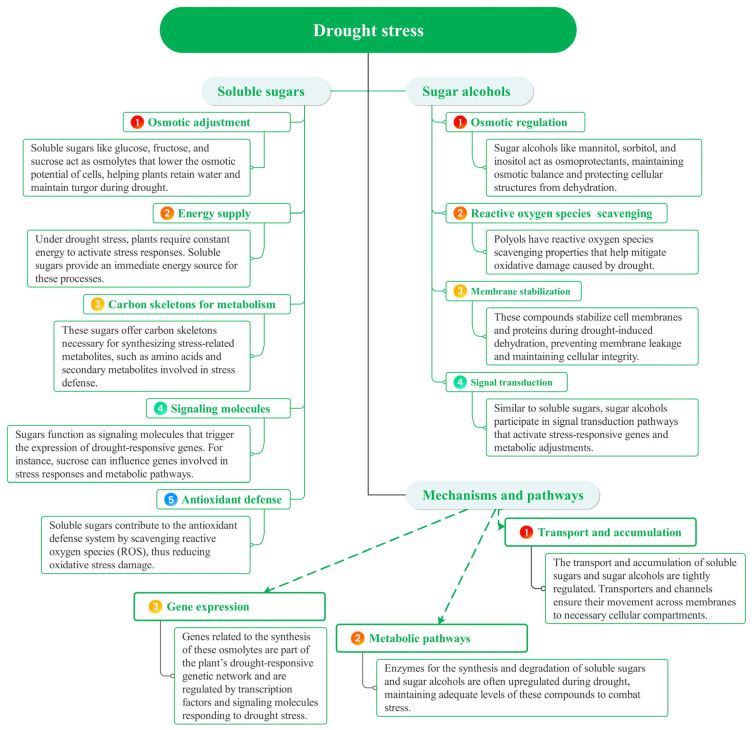
Soluble sugars and sugar alcohols play crucial roles in plant responses to drought stress.

Crops in arid regions experience significant drought stress, underscoring the necessity of understanding the mechanisms that govern drought resistance to enhance crop yields and promote sustainable agriculture. A primary strategy employed by these crops involves the regulation of osmotic adjustment substances, which helps to maintain the water balance both within and outside of cells [31]. In response to drought stress, osmotic regulators, such as betaine and proline, accumulate, aiding in the preservation of cell turgor and the stabilization of enzyme activity and protein structures [32]. Furthermore, small molecule osmotic regulators, including soluble sugars and sugar alcohols, play a crucial role in the drought response (Figure 3).

The antioxidant defense system plays a crucial role in mitigating the oxidative damage caused by drought. Antioxidant enzymes, such as superoxide dismutase (SOD), peroxidase (POD), and ascorbate peroxidase (APX), exhibit increased activity under drought stress, working collaboratively to scavenge reactive oxygen species and reduce membrane lipid peroxidation [33]. Furthermore, plant hormones are essential for drought stress signaling and the regulation of drought responses [34]. Abscisic acid (ABA) serves as a key hormone that enhances drought tolerance by regulating stomatal closure and inducing the expression of osmotic adjustment genes. The exogenous application of ABA has been shown to significantly improve drought tolerance in crops such as wheat [35] and maize [36]. Other hormones, including cytokinins, gibberellins, and brassinosteroids, also participate in drought signaling pathways. However, their specific roles require further investigation [37].

Transcriptomic and proteomic studies reveal significant alterations in the expression levels of numerous drought-related genes and proteins during drought stress [38]. These genes play crucial roles in osmotic adjustment, antioxidant defense, protein synthesis, degradation, and signal transduction, collectively forming a complex regulatory network that governs the plant’s response to drought [39]. A detailed functional analysis of these key genes can illuminate the molecular mechanisms underlying drought resistance, thereby providing valuable insights for the breeding of drought-resistant varieties.

### 2.4. Molecular Biological Mechanism of Drought Tolerance in Crops

Drought resistance in crops is a complex trait that is regulated by multiple molecular mechanisms. A primary response to drought stress involves changes in the expression of stress-related genes. Under drought conditions, the genes associated with osmotic regulation and cellular protection, such as late embryogenesis abundant (LEA) protein genes and osmolyte synthesis enzyme genes, are upregulated, thereby enhancing drought tolerance in crops [40]. Furthermore, transcription factors play a crucial role in regulating the expression of these drought-responsive genes. Families of transcription factors, including dehydration-responsive element-binding (DREB) proteins [14], ABA-responsive element-binding (AREB) proteins [41], and NAM, ATAF, and CUC (NAC) proteins [42], bind to the promoter regions of the target genes, activating their expression to improve drought resistance.

In addition to transcriptional regulation, post-transcriptional mechanisms, particularly those mediated by microRNAs (miRNAs), play a crucial role in enhancing crop drought resistance. miRNAs are endogenous non-coding RNAs that regulate gene expression by binding to target mRNAs and directing the RNA-induced silencing complex (RISC) to either degrade the mRNA or inhibit its translation [43]. Specific drought-responsive miRNAs, such as miR398 and miR169, have been identified [44]. These miRNAs negatively regulate the genes associated with antioxidant enzymes and transcription factors, thereby indirectly influencing the expression of drought-related genes and playing significant roles in stress responses [45]. Furthermore, other non-coding RNAs, including long non-coding RNAs (lncRNAs) and circular RNAs (circRNAs), also contribute to drought responses. However, their functions require further investigation [46].

Post-translational modifications (PTMs) of proteins play a critical role in regulating drought resistance in crops. The key PTMs involved in stress response pathways include phosphorylation, ubiquitination, and glycosylation [47]. For instance, members of the SnRK2 (SNF1-related protein kinase 2) family phosphorylate downstream proteins, such as transcription factors and ion channel proteins, thereby modulating their activity and influencing the expression of drought-related genes [48]. The dynamic interplay between kinases and phosphatases is essential for maintaining appropriate phosphorylation levels, which, in turn, supports stress adaptation. Furthermore, ubiquitin-mediated protein degradation is crucial for removing damaged proteins and reorganizing the proteome, thereby contributing to homeostasis under drought stress [49].

Signal transduction pathways are central to the initiation of the molecular mechanisms underlying drought resistance. Upon detecting drought signals, crops transmit these signals from the cell surface to intracellular compartments via second messengers, such as calcium ions, reactive oxygen species (ROS), and phospholipids [50]. These messengers trigger cascades that activate the effector molecules responsible for drought tolerance [51]. The ABA pathway plays a pivotal role in this process. Under drought conditions, increased levels of ABA bind to PYR/PYL/RCAR receptors, alleviating the inhibition of SnRK2 kinases by PP2C phosphatases [52]. Activated SnRK2 kinases subsequently phosphorylate AREB transcription factors, thereby promoting the expression of drought-responsive genes [53]. Additionally, the mitogen-activated protein kinase (MAPK) cascade and calcium-dependent protein kinase (CDPK) pathways contribute to drought responses, with the cross-talk between these pathways forming a complex regulatory network [48].

In summary, drought resistance in crops results from the coordinated interaction of gene expression, miRNA-mediated regulation, post-translational modifications, and signal transduction pathways. These molecular mechanisms form a foundation for the genetic enhancement of drought resistance. Future research should focus on further elucidating these regulatory mechanisms and identifying additional drought-responsive genes, miRNAs, and signaling components. The integration of advancements in molecular biology with traditional breeding techniques will be essential for developing drought-tolerant, water-efficient crops, thereby accelerating sustainable agricultural development in arid regions and ensuring food security.

### 2.5. Traditional and Modern Cross-Breeding Techniques

Crop breeding is a critical avenue for enhancing both yield and quality. The integration of traditional breeding methods with modern molecular techniques can accelerate the selection of superior varieties that exhibit both drought resistance and high yield. Conventional breeding methods, such as hybrid breeding and mutation breeding, primarily rely on artificial hybridization and mutagenesis to create new genetic variations, followed by a systematic selection to obtain superior varieties [54]. In contrast, modern breeding incorporates biotechnological approaches, including molecular marker-assisted selection and transgenic breeding, which enhance the precision and efficiency of trait improvement [55]. By utilizing the linkage between molecular markers and the genes associated with target traits, the early screening of hybrid offspring significantly improves selection efficiency and breeding progress [56]. Furthermore, genetic engineering techniques facilitate the introduction of exogenous drought-resistant genes into crop genomes, directly resulting in the development of new germplasms with enhanced drought resistance [5].

Conventional breeding that utilizes heterosis (hybrid vigor) is an effective strategy for crop improvement. By systematically selecting parent lines and designing scientifically informed hybrid combinations, researchers can produce first-generation hybrids that exhibit superior growth rates, yields, quality, and resistance [57]. For instance, the successful development of indica–japonica hybrid rice through heterosis has resulted in significant improvements in both yield and quality [58]. Additionally, distant hybridization enhances the genetic diversity of crops by introducing interspecific differential genes into cultivated species, thereby providing new germplasm resources for the enhancement of complex traits, such as drought resistance and water efficiency [59]. However, distant hybridization often encounters challenges, including hybrid incompatibility and offspring sterility, which necessitate the application of biotechnological methods, such as embryo rescue, to improve success rates.

Modern molecular breeding techniques provide innovative perspectives and methodologies for investigating crop drought resistance. The isolation, identification, and functional verification of drought-related genes are the foundational elements of molecular breeding aimed at enhancing drought resistance. By utilizing genomic tools such as gene chips and next-generation sequencing, researchers can analyze the molecular regulatory networks associated with crop drought resistance at multiple levels, including transcriptomics and proteomics, thereby revealing key drought-resistant genes [60]. Through transgenic and gene-editing technologies, precise enhancements of these genes can be achieved, leading to the development of new drought-resistant germplasm. Furthermore, genome-wide association studies (GWAS) and multi-omics association analyses offer valuable insights for the fine mapping and in-depth examination of drought-resistant genes. When combined with a phenomics analysis, these approaches deepen our understanding of the genetic regulation of drought traits, fostering theoretical advancements in drought-resistant breeding. The construction of a comprehensive diversity database of crop drought-related genes, along with the systematic identification and exploitation of superior allele variations, will facilitate the aggregation and enhancement of drought-resistant genes, as well as support molecular design breeding.

The integration of conventional and modern breeding technologies, along with the optimization of breeding systems and technical schemes, is essential for accelerating the development of new drought-resistant and water-efficient crop varieties. Local varieties in various drought-prone regions possess a wealth of drought-resistant gene resources, having evolved unique mechanisms for drought resistance through prolonged environmental adaptation [61]. These valuable local germplasms contain key genes that can enhance crop drought resistance and should be systematically developed and utilized. Furthermore, drought-resistant genes from wild relatives represent excellent germplasm resources that ought to be incorporated into the genetic background of cultivated species through distant hybridization and other techniques. Additionally, enhancing multiple target traits, such as drought resistance, water efficiency, quality, and high yield, to create new varieties with superior agronomic characteristics will be a significant focus in crop breeding. Achieving this goal necessitates an interdisciplinary approach that integrates physiology, genetics, genomics, and other fields to thoroughly analyze the genetic mechanisms underlying important crop traits, innovate breeding theories and methodologies, and comprehensively strengthen the technological support capacity of modern agriculture.

## 3. Improved Crop Stress Resistance

### 3.1. Improved Salt and Alkali Resistance

Improving and restoring saline–alkali soils in arid regions is crucial for enhancing agricultural productivity. While traditional chemical amendments, such as gypsum and sulfur, provide rapid results, they can also lead to soil compaction and secondary salinization [62]. In contrast, bio-organic fertilizers are environmentally friendly and offer long-lasting benefits. These fertilizers increase soil organic matter, enhance both the physical and chemical properties of the soil, and promote the leaching of salts [63]. Research indicates that increasing the soil organic matter content above 1.5% can significantly improve crop drought resistance [64]. Furthermore, the application of compound fertilizers containing humic acid and amino acids can accelerate soil maturation and enhance soil structure [65]. Practices such as returning crop residues to the field and planting green manure can further bolster soil fertility and water retention, thereby aiding in the mitigation of salinization.

Selecting and breeding salt-tolerant crop varieties represents an effective strategy for optimizing the use of saline–alkali soils. Systematic surveys and evaluations of germplasm resources can facilitate the development of new crop varieties that exhibit enhanced salt tolerance and high yields. Particular emphasis should be placed on local salt-tolerant crops, such as pomegranates and goji berries, while integrating traditional breeding methods with modern biotechnologies to cultivate high-yield, high-quality salt-tolerant varieties of major crops, including rice and wheat [66]. Furthermore, research on effective cultivation techniques and integrated management strategies for salt-tolerant crops should be expanded to establish high-yield models that encompass variety improvement, fertilizer, and water management.

Rhizosphere microorganisms play a crucial role in enhancing saline–alkali soils. The inoculation of beneficial bacteria, such as nitrogen-fixing and phosphate-solubilizing bacteria, significantly improves nutrient cycling and transformation within the rhizosphere, thereby increasing fertilizer efficiency [67]. The selection and acclimatization of effective microbial strains to develop salt-tolerant, rhizosphere growth-promoting agents can substantially enhance both crop yield and quality. Furthermore, inoculating crops with symbiotic microorganisms, including arbuscular mycorrhizal fungi, can strengthen crop resistance to salt–alkali stress [68]. Enhancing fundamental research on the interactions and regulatory pathways between rhizosphere microorganisms and crops will facilitate the development of innovative microbial fertilizers and advance technologies for the restoration of rhizosphere microecology.

**Figure 4:**
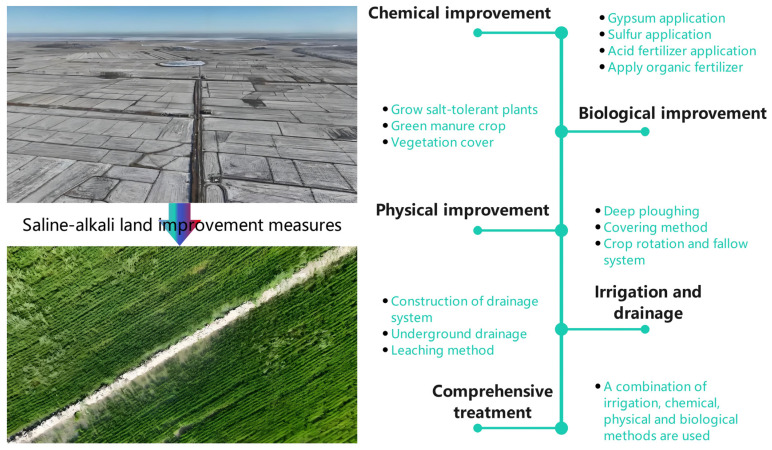
The main measures of saline–alkali land improvement.

The ecological restoration of saline–alkali land represents a complex system that requires integrated management tailored to local conditions. For severely salinized soils, strategies such as soil replacement, sand mulching, and biological salt control, when combined with agronomic practices and the planting of trees and grasses, can progressively improve soil salinity (Figure 4). In the case of mildly to moderately saline–alkali soils, cultivating salt-tolerant crops and optimizing planting patterns can facilitate the movement of water and salts within the soil. Furthermore, enhancing farmland infrastructure through the strategic placement of drainage ditches, subsurface pipes, and anti-seepage channels can significantly increase soil desalination capacity. Additionally, precision irrigation management that utilizes advanced sprinkler and drip irrigation technologies enables efficient water and fertilizer application, reduces agricultural water consumption, and helps maintain a balance between the soil water and salt levels.

### 3.2. Disease Resistance and Pest Cultivation in Arid Areas

Pests and diseases pose a significant threat to crop yield and quality, representing one of the primary limitations to agricultural development in arid regions. Biological control, which employs natural enemy insects and microorganisms, offers an environmentally sustainable approach to managing these challenges. Recent advancements in biocontrol agents include the utilization of fungi, such as *Beauveria bassiana* and *Metarhizium anisopliae,* for controlling vegetable pests, the release of *Trichogramma* wasps to combat cotton bollworms, and the application of *Bacillus thuringiensis* for managing lepidopteran pests [69]. These agents are highly specific and have minimal environmental impact, making them essential components of integrated pest management (IPM). Furthermore, breeding crops for pest and disease resistance is crucial for enhancing resilience. Conventional breeding has produced several resistant varieties, while biotechnological techniques, including marker-assisted selection and genetic engineering, enable the precise introduction of resistance genes, resulting in varieties that combine resistance with high yield and quality [70].

Integrated pest management (IPM) combines agricultural, physical, chemical, and biological strategies to control pests and diseases within economic thresholds, thereby optimizing economic, ecological, and social outcomes through sustainable practices [71]. In IPM systems, the patterns of pest and disease occurrence are analyzed to identify optimal control timings and economic thresholds, employing strategies such as timely regulation, scientific fertilization, physical trapping, biological control, and selective pesticide application [72]. For instance, in the IPM approach for wheat stripe rust, the implementation of resistant varieties, timely sowing, appropriate planting density, and field sanitation act as preventive measures that are complemented by chemical control to effectively manage disease outbreaks [73]. Similarly, in the IPM strategy for the cotton bollworm, a combination of light traps, sex pheromone traps, biological control, and judicious chemical application reduces pesticide usage while maintaining pest populations at manageable levels [74]. Establishing an IPM system that integrates multiple control methods is crucial for managing pests and diseases while minimizing pesticide application, thus representing the future of plant protection [75].

**Figure 5:**
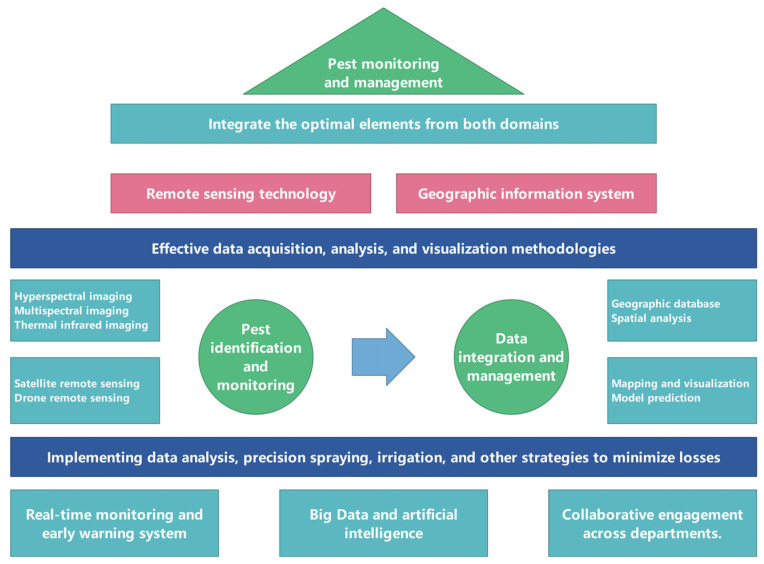
The practical application of remote sensing and geographic information systems to monitor and manage pests and diseases in large areas.

With advancements in modern information technology, the construction of pest and disease monitoring and early warning systems utilizing multi-source data has become feasible, providing scientific decision-making tools for agricultural management. Remote sensing and geographic information systems (GIS) facilitate large-scale monitoring of pest and disease outbreaks by extracting data, such as vegetation indices and soil moisture, to assess damage severity (Figure 5). Tools like light traps and sex pheromone traps gather real-time data on pest species and populations, which, when integrated with microclimate data such as temperature and humidity, enable precise pest monitoring [76]. Big data analysis can subsequently generate timely alerts regarding the timing, location, and severity of pest outbreaks, assisting farmers in implementing preventive measures [77]. For instance, the United States has amassed decades of pest and disease data to establish a modern plant protection information system that supports regional control strategies [78]. Similarly, China has developed effective early warning systems for significant pests and diseases, including wheat stripe rust and rice sheath blight [79]. The development of pest and disease monitoring and early warning systems is crucial for tracking major outbreaks, optimizing the allocation of plant protection resources, and advancing the field of plant protection science.

## 4. Intelligent Agriculture Produces Efficiently

### 4.1. Precision Agriculture Management System

Precision agriculture management systems represent a crucial strategy for enhancing the quality and efficiency of agriculture in arid regions. By utilizing various sensor devices, real-time monitoring and data collection of environmental parameters, such as soil moisture, temperature, and nutrient levels, can be effectively achieved, providing a reliable foundation for informed agricultural decision-making [80]. Data from these sensors is transmitted to a centralized hub via wireless networks, where machine-learning algorithms and agricultural knowledge bases are employed to assess crop growth and forecast future conditions. This information is subsequently used to develop optimized field management plans for irrigation and fertilization [81]. Compared to traditional farming methods, digital precision agriculture systems significantly improve resource utilization by enabling precise control over agricultural inputs, resulting in substantial savings in water and fertilizer, while ensuring high yields and maintaining crop quality.

In addition to the previously mentioned systems, participatory, remote-sensing-fed approaches have been widely adopted in precision agriculture. For instance, Manna Irrigation, IRMA_SYS, and IrriSAT exemplify systems that do not require sensors to be installed in each field, allowing for easy and cost-effective application across large areas. These systems leverage the capabilities of remote sensing and big data to monitor and analyze crop conditions, enabling farmers to make informed decisions regarding irrigation, fertilization, and other farming practices. Consequently, these approaches enhance resource utilization efficiency and improve agricultural productivity.

Blockchain technology offers significant opportunities for improving the traceability of agricultural products and supply chain management. By recording farming operations, environmental data, and input usage on a blockchain, an immutable and fully traceable record of agricultural production is created [82]. Consumers can access detailed information about the planting process by scanning a product’s QR code, which enhances brand reputation and fosters consumer trust in high-quality agricultural products [83]. Furthermore, blockchain technology contributes to increasing the added value of agricultural products, streamlining production and sales channels, and boosting farmers’ income [84]. Simultaneously, the integration of agricultural big data and smart agricultural management platforms is advancing. The vast amounts of data collected by agricultural IoT systems in real time provide a foundation for precision agriculture, while big data analysis optimizes industry layout and supports macro-level policy formulation.

The development of smart agriculture ecosystems relies on the interconnection and sharing of multi-source, heterogeneous data, as well as the innovative application of agricultural artificial intelligence technologies. Precision agriculture, driven by the integration of information technology with agricultural production, is transforming traditional farming methods by transitioning from experience-based management to data-driven, intensive farming practices. This shift opens new pathways for sustainable and high-quality agricultural development. As next-generation technologies, such as 5G and artificial intelligence, continue to advance, precision agriculture management systems are anticipated to become increasingly intelligent and automated, thereby propelling the modernization of agriculture and enhancing national food security.

### 4.2. Application of Agricultural Internet

The application of IoT technology in agriculture is expanding rapidly, particularly in arid regions. By employing intelligent sensors for field environment monitoring and automated irrigation control, the efficiency of water resource utilization can be significantly enhanced [85]. Various sensors, including temperature, humidity, and soil moisture sensors, are deployed in fields to collect real-time data on microclimate conditions and soil moisture levels. These data are transmitted via wireless communication networks to cloud platforms for analysis. Through big data analysis, irrigation systems can automatically adjust the timing and amount of water based on crop water requirements and soil conditions, thereby enabling precise irrigation [86].

Additionally, the agricultural IoT can be integrated with mobile internet technology, enabling remote monitoring and control. Farmers can assess field conditions and manage irrigation systems from a distance using mobile applications. Furthermore, the synergy between agricultural robotics and IoT technology is pivotal in the advancement of intelligent agriculture [87]. Agricultural robots can autonomously plan operational paths based on environmental data collected through the IoT, executing tasks such as precise seeding, fertilization, and pesticide application. This integration reduces labor intensity while enhancing accuracy [88]. Some robots are also equipped with multispectral imaging devices to monitor crop growth, thereby providing farmers with data-driven support for agronomic decision-making.

The environmental monitoring capabilities of the agricultural IoT not only facilitate irrigation control but also enable the early detection of pest threats, thereby aiding in disaster prevention and mitigation [89]. The deployment of video surveillance and pest-monitoring lamps in fields allows for the remote observation of pest activity. Upon detecting pests, the system sends alerts to farmers, accompanied by recommended control measures. Furthermore, the meteorological data collected by agricultural IoT systems, such as sunlight and precipitation, can be utilized to issue early warnings and forecasts for agricultural meteorological disasters, including droughts, floods, and hailstorms, thereby assisting farmers in taking timely preventive actions [90].

The integration of agricultural IoT technology with digital and information systems is propelling the modernization of agriculture [91]. However, challenges remain, including high costs, inconsistent standards, and limited compatibility across data platforms. Future efforts should focus on addressing these fundamental technological barriers, establishing unified data standards and protocols, and promoting the development and adoption of essential IoT products in agriculture. These initiatives will expand the benefits to a larger number of farmers, enhance agricultural productivity, and support sustainable, high-quality agricultural development.

### 4.3. Remote-Sensing Technology and Crop Monitoring

The application of remote-sensing technology in agriculture is becoming increasingly widespread, particularly for monitoring crop growth. By utilizing multispectral imagery from satellites and drones, farmers can observe crops in real-time, thus providing valuable scientific support for agricultural management [92]. High-resolution satellite imagery enables the calculation of vegetation indices, such as the Normalized Difference Vegetation Index (NDVI) and Enhanced Vegetation Index (EVI), which quantitatively assess crop health and estimate yields [93]. Additionally, thermal infrared remote sensing can monitor crop canopy temperature, providing insights into water status and facilitating early drought warnings [94]. Active remote-sensing technologies, such as LiDAR and synthetic aperture radar (SAR), also offer innovative methods for acquiring three-dimensional data on crop structure and soil moisture [95].

With the advancement of drone remote-sensing technology, low-altitude observation of farmland using drones equipped with multispectral cameras has become increasingly routine. Drone remote sensing offers advantages, such as flexibility, short revisit cycles, and high resolution, which make it particularly suitable for fine-scale monitoring at the farm level. By obtaining centimeter-level resolution images in the visible and near-infrared spectra, and employing machine-learning algorithms, it is possible to extract crop planting areas, assess crop vigor, and detect pests and diseases [96]. Some studies have integrated drone remote sensing with ground surveys to establish a comprehensive crop monitoring system, thereby achieving accurate monitoring and forecasting of crop growth on a regional scale [97]. However, drone remote sensing also has limitations, including short flight durations and light payload capacities, which necessitate its use in conjunction with satellite remote sensing for large-area crop monitoring [98].

Accurate positioning is essential for farm-scale crop monitoring using remote-sensing technologies. Global navigation satellite systems (GNSS), including GPS and BeiDou, provide the precise positioning necessary for remote-sensing data collection. By integrating GNSS receivers into platforms such as drones and mobile devices, centimeter-level precision can be achieved, thereby ensuring the accuracy of the geographic coordinates of the remote-sensing data collected [99]. This advancement significantly enhances data processing, analysis, and mapping capabilities. Furthermore, high-precision positioning supports applications such as the automated navigation of agricultural machinery and precision fertilization, promoting greater automation in agricultural operations.

The integration of remote-sensing technology into crop monitoring represents a significant trend in contemporary agriculture. Agricultural remote sensing is evolving towards multi-platform, multi-scale, and multi-temporal systems, which continuously expand data sources and analytical methods. In the future, hyperspectral and high-resolution satellite data are expected to become more accessible, while the applications of drone remote sensing will broaden. The incorporation of artificial intelligence in the analysis of remote-sensing data will further enhance the intelligence and precision of crop monitoring. Consequently, remote-sensing technology is poised to play an increasingly vital role in promoting agricultural modernization and improving food security.

## 5. Water Resources Efficient Utilization Technology

### 5.1. Farmland Water Resources Management

The cyclic utilization and reuse of water resources are essential for sustainable agricultural development in arid regions. Properly treated agricultural wastewater can be repurposed for irrigation, thereby alleviating water scarcity and minimizing pollution from wastewater discharge. In developed countries, recycled water is extensively used for irrigation. For instance, half of Israel’s irrigated farmland relies on recycled water [100]. However, the reuse of agricultural wastewater necessitates the integration of sewage treatment technologies. Traditional sewage treatment processes, such as the activated sludge method, are primarily designed for industrial and domestic wastewater and are less effective for treating agricultural wastewater [101].

Rainwater is a valuable resource in arid agriculture, and its efficient utilization is crucial for enhancing crop yields and improving the ecological environment. Rainwater harvesting facilities should be designed according to local climate, terrain, and crop water requirements [102]. Structures such as ponds, reservoirs, and underground tanks are vital for effective rainwater collection and storage. Optimizing the ratio between catchment and irrigation areas, along with implementing soil improvement measures to enhance infiltration and water retention, can minimize rainwater runoff loss [103]. In China’s Loess Plateau, strategic crop placement based on the natural terrain contributes to the establishment of stable and efficient farmland ecosystems [104]. Additionally, practices such as intercropping and mixed cropping can further enhance land use efficiency and bolster agricultural production diversity and resilience [105].

Rooftop rainwater collection is an effective method for optimizing the use of rainwater. By installing collection systems on rooftops and directing runoff into storage tanks or underground reservoirs, losses of rainwater can be minimized. The relatively high quality of water collected from rooftops makes it suitable for crop irrigation and even domestic use, thereby helping to alleviate water shortages in arid regions. Effective runoff management is crucial for maximizing rainwater utilization. Techniques such as terracing, contour planting, and furrow planting can effectively retain runoff, reduce soil erosion, and enhance infiltration [106]. In areas where runoff tends to concentrate, the construction of ponds and sedimentation tanks can mitigate flood peaks and extend the availability of rainwater. Additionally, planting trees and vegetation around fields slows runoff, promotes infiltration, and improves microclimatic conditions.

Optimizing irrigation scheduling in arid regions is essential for enhancing water use efficiency and improving crop yields [107]. This necessitates an interdisciplinary approach that integrates modern technology with traditional agricultural practices. Future initiatives should prioritize foundational research in agricultural hydrological processes and crop growth models. The advancement of intelligent irrigation control and scheduling technologies, alongside water-saving measures such as agricultural water pricing reforms, is critical for promoting precision irrigation. Raising farmers’ awareness and skills through demonstration bases and technical training can facilitate the adoption of these advanced irrigation methods. Achieving sustainable agricultural development in arid regions requires a holistic approach that encompasses technological, managerial, and policy innovations.

### 5.2. Agricultural Water-Saving Irrigation Technology

Drip and sprinkler irrigation are two efficient, water-saving technologies widely employed in arid agricultural production. Drip irrigation delivers water directly to crop roots through pipelines, thereby minimizing evaporation and leakage losses and achieving an irrigation efficiency exceeding 90% [108]. Sprinkler irrigation, on the other hand, sprays water into the air under high pressure, creating fine droplets that moisten both the soil and crops, thus providing excellent irrigation uniformity and significant water-saving benefits. The selection of an irrigation method and parameters is influenced by several factors, including the crop type, growth stage, and soil characteristics [109]. Adaptive control systems can modify irrigation strategies in real time based on soil moisture levels and meteorological conditions, facilitating precise irrigation and intelligent management.

**Figure 6:**
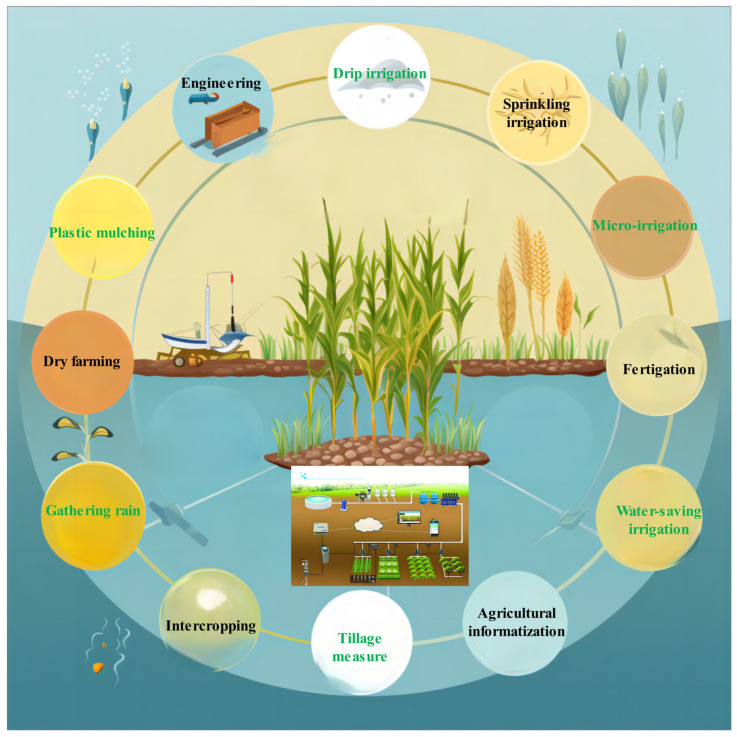
Improve the efficiency of water resources utilization and reduce agricultural water use through agricultural water-saving technology to ensure that crops can grow and develop normally under the condition of limited water resources.

Compared to traditional surface irrigation methods, micro-irrigation and subsurface irrigation systems significantly conserve water (Figure 6). Micro-drip irrigation devices deliver water precisely near crop roots, utilizing soil capillary action to evenly distribute moisture within the root zone [110]. This approach markedly reduces water consumption, requiring only one-fifth to one-sixth of the water used in surface irrigation, while also minimizing evaporation and leakage losses, thereby enhancing water use efficiency [111]. Furthermore, by dissolving fertilizers in the subsurface irrigation system and applying them directly to crop roots, fertilizer use efficiency can be improved by approximately 30%, thus achieving the objectives of conserving water, fertilizer, and labor [112].

Root zone irrigation technology is fundamental to micro-irrigation and subsurface irrigation systems. By directly delivering water and nutrients to the root zone, it significantly enhances water and nutrient use efficiency, thereby promoting crop growth [113]. The movement of soil moisture is crucial for developing irrigation schedules and is influenced by factors such as soil texture, structure, and organic matter content. Coarse sandy soils exhibit high water conductivity but poor water retention, necessitating small and frequent irrigation, whereas clay soils demonstrate low water conductivity but excellent water retention, permitting longer intervals between irrigations [114]. Agronomic practices, such as deep plowing, no-till farming, and straw mulching, have a significant impact on soil infiltration performance and water retention capacity [115].

Economic benefit analysis is crucial for evaluating irrigation technologies. While drip irrigation provides significant water-saving and yield-increasing advantages, its initial investment is relatively high. In contrast, sprinkler irrigation requires a lower initial investment but incurs higher energy consumption and operating costs [116]. A comprehensive evaluation should take into account the economic value of crops, irrigation expenses, and benefit ratios. In arid and semi-arid regions with limited water resources, the benefits of drip irrigation are increasingly recognized [117]. However, long-term agricultural irrigation must also consider factors such as resource allocation, ecological impacts, and input–output ratios to achieve multi-objective optimization.

### 5.3. Conservation Tillage

Conservation tillage is a crucial strategy for improving the quality and efficiency of agriculture in arid regions. The maintenance of healthy topsoil is essential for sustainable agricultural production and human survival. Conservation tillage techniques play an indispensable role in controlling wind and water erosion, reducing dust pollution, and enhancing soil fertility and drought resistance [118]. Key components of conservation tillage include no-tillage and water-conserving tillage methods. These techniques prevent the surface crusting caused by raindrop impact, reduce the moisture loss associated with excessive tillage, maintain an optimal soil structure, and enhance the utilization of fertilizers applied to the tillage layer [119]. By retaining crop residues and utilizing cover materials to capture rainfall, soil organic matter is increased, thereby sustaining the long-term fertility of the soil [120].

The application of organic mulches represents a key technology in conservation tillage. Decomposed mulch not only increases soil organic matter but also sustains soil fertility. The combined effects of residue retention and mulching significantly enhance soil water-holding capacity and fertility [121]. Additionally, crop rotation and intercropping serve as important components of conservation tillage systems. Effective crop rotation disrupts monoculture practices, controls pests and diseases, and promotes soil health. Intercropping enhances land use efficiency, increases biodiversity on farmland, and capitalizes on the complementary relationships among different crops [122]. Furthermore, incorporating nitrogen-fixing crops such as legumes into rotation systems reduces the need for chemical fertilizers and improves the efficiency of nitrogen fertilizer usage.

Integrating biological control techniques with tillage practices represents an effective strategy for achieving sustainable agricultural development. The use of biological pesticides and natural predators not only reduces reliance on chemical pesticides but also minimizes the risks of environmental pollution. Furthermore, conservation tillage fosters soil microbial diversity and activity, thereby enhancing the soil’s capacity for self-repair. By implementing an integrated biological–tillage management approach, it is possible to safeguard the safety and quality of agricultural products while concurrently maintaining the ecological balance of farmland.

### 5.4. Soil Water Conservation and Improvement

In semi-arid regions, enhancing water use efficiency through environmental control is essential for increasing agricultural yields. The application of soil water-absorbing and retaining materials can significantly improve the soil’s water-holding capacity [123]. By utilizing the low hydraulic head from on-site water storage ponds, irrigation water can be delivered directly to the root zone via buried multi-level pipelines. This method capitalizes on soil capillary action to facilitate precise irrigation, thereby enhancing water use efficiency. Furthermore, it mitigates soil surface crusting caused by rainfall, reduces moisture loss due to excessive tillage, and sustains a favorable soil structure [124].

Increasing soil organic matter is essential for enhancing soil quality in arid regions, as it directly impacts both crop yield and quality. While traditional methods, such as applying farmyard manure and returning straw to the field, have some benefits, they are often challenging to implement and provide limited improvements. In contrast, shredding pruned fruit tree branches and composting them in situ for over two years can substantially increase soil organic matter content [125]. This composting process also fosters the proliferation of earthworms, thereby enhancing the overall soil biological activity.

Deep loosening tillage is a crucial strategy for enhancing soil fertility in arid regions. In comparison to traditional multiple tillage and deep tillage methods, no-till and conservation tillage effectively reduce soil moisture evaporation, improve fertilizer utilization, and promote the formation of soil aggregates [119]. The adoption of practices such as straw mulching, stubble retention, no-till seeding, and chemical weeding decreases tillage frequency, obstructs soil capillaries, inhibits soil moisture evaporation, and mitigates the effects of drought [126]. Furthermore, soil structure amendments, including the application of gypsum and aluminum sulfate as soil conditioners, can enhance the soil aggregate structure and increase the water-holding capacity [127].

## 6. Summary and Prospect

In arid regions, agriculture has experienced significant advancements in crop breeding, production efficiency, water-saving technologies, and food processing. Modern biotechnologies, including genetic engineering and marker-assisted selection, have facilitated the development of drought- and salt-tolerant crop varieties, thereby enhancing the genetic resources available for sustainable agriculture. Furthermore, the utilization of native crops and wild germplasm has increased genetic diversity and improved stress resistance. The implementation of smart agriculture and water-saving technologies has revolutionized production efficiency and resource management. Techniques such as remote sensing, the IoT, and big data integration enable the real-time monitoring of soil moisture and crop growth. Additionally, conservation tillage and advanced irrigation methods improve water use efficiency, while controlled-environment agriculture optimizes land utilization and mitigates drought stress. Collectively, these advancements have laid a foundation for sustainable agricultural development in arid regions, contributing to regional food security and economic growth. However, ongoing research and technological innovation remain crucial for effectively addressing the challenges faced in dryland agriculture.

Precision Agriculture 3.0 utilizes advanced technologies to facilitate intelligent and precise agricultural management. Real-time data capture through sensors and machine-learning algorithms optimizes irrigation and fertilization, thereby reducing costs and enhancing efficiency. Furthermore, automated navigation and variable-rate fertilization enhance the precision of agricultural machinery. Gene-editing technologies, such as CRISPR/Cas, offer transformative tools for crop breeding by enabling precise modifications to target genes, which accelerates the development of stress-resistant varieties. When integrated with conventional breeding and omics technologies, gene editing expedites advancements in crop breeding aimed at improving climate resilience and ensuring food security. Smart agricultural ecosystems merge information technology with production, management, and services. Real-time field monitoring and intelligent platforms facilitate precise decision-making. Traceability systems enhance food safety, while supply chain optimization and e-commerce contribute to the modernization of agriculture.

Climate change poses significant threats to food production. Coordinated efforts in breeding, cultivation, and management are essential for developing resilient agricultural systems. Key components of this endeavor include the development of heat-, drought-, and salt-tolerant germplasm, the implementation of water-saving practices, the establishment of efficient irrigation systems, and preparation for potential disasters. Embracing green development, promoting technological innovation, and integrating sustainability strategies are crucial for mitigating the impacts of climate change and ensuring reliable food supplies in arid regions.

## Data Availability

No new data were created or analyzed in this study.
